# Service user involvement in the coproduction of a mental health nursing metric: The Therapeutic Engagement Questionnaire

**DOI:** 10.1111/hex.12526

**Published:** 2017-07-12

**Authors:** Mary Chambers, Susan McAndrew, Fiona Nolan, Ben Thomas, Paul Watts, Xenya Kantaris

**Affiliations:** ^1^ Faculty of Health, Social Care and Education Kingston University and St George's, University of London London UK; ^2^ School of Nursing, Midwifery, Social Work & Social Sciences University of Salford Manchester UK; ^3^ Florence Nightingale Foundation Chair in Clinical Mental Health Nursing Practice Research University of Essex School of Health and Human Sciences UK; ^4^ Department of Health Strategy and External Relations Directorate London UK; ^5^ Community Mental Health Nursing Somerset Partnership NHS Foundation Trust Bridgwater UK

**Keywords:** engagement, mental health services, nursing metric, research, service user

## Abstract

Service users’ involvement in mental health service research is increasingly acknowledged as important, yet, whilst involving users of mental health services as research participants is commonplace, seeking out their experience and indeed their “expertise” to facilitate the development of tools to be used within mental health services is in its infancy. This article describes the involvement and views of service users in the development of a nursing metric—the Therapeutic Engagement Questionnaire. It presents their role in the three stages of development: generation, statement reduction and authentication.

## 1 INTRODUCTION

### 1.1 The role of the service user

There are many ways to describe a service user.^1^ In this instance, a service user is defined as a person who is accessing or who has accessed inpatient mental health care services. Service user involvement has been defined by one UK charity as the “active participation of a person with lived experience of mental distress in shaping their personal health plan based on their knowledge of what works best for them. It's about people asserting their perspectives and being heard in their own right. It is about being active, not passive.”^2^

Increasingly, health‐care research has involved service users as participants rather than researchers. This article describes the involvement of service users in the development of the Therapeutic Engagement Questionnaire (TEQ), specifically in generating the potential statements for the tool, its pre‐testing and revisions. Whilst the project was initially researcher‐led, comprehensive service user engagement was evident throughout the stages described. In this way, the service users’ voice was “heard” and their perspective respected, despite their absence from the original research team.

Different perspectives and standpoints inevitably produce different ways of understanding and interpreting phenomena.^1^ Although different terms exist to describe people who use health and social care services, for example clients and patients, we have chosen to use the term service user. This seems more appropriate as “client” seems cold and has the overtone of receiving services rather than an equal relationship with health and social care practitioners.

### 1.2 The importance of service user involvement

For health and social care research to be of real benefit to service users, professionals must strive to involve them in setting the questions to which they are seeking answers. Traditionally, outcomes in health and social care have largely been determined by objective evaluations such as costs and benefits of care. However, it has become increasingly clear that the perspective of the service users is also a critical variable.^3^ It is now increasingly common, and indeed sometimes a requirement, to include reports from a service user's perspective in evaluations of health and social care interventions/services. This approach has the merit of potentially empowering service users.^4^ Health and social care researchers would deny themselves a key source of information if service users were not involved in some aspect of their research plans;^5^ service user involvement needs to be an integral part of mental health services and organizations for them to be truly effective.^6^ However, such involvement must be “active involvement,” whereby service users work collaboratively with researchers in making decisions about what questions are asked and how these are translated into meaningful research projects.^7^ Evidence shows that active service user involvement throughout the research process results in outcomes that are relevant and useful.^4^ Likewise, when service users’ knowledge is recognized, valued and taken seriously, it is likely to be an empowering experience.^2^

### 1.3 The goal

Since the publication of Peplau's seminal work^8^ which emphasized the primacy of the nurse‐patient relationship, therapeutic engagement has been considered the crux of psychiatric nursing.^9^ Current evidence suggests that therapeutic engagement is beneficial and is of significant clinical importance.^10^, ^11^ Indeed, service users value positive attitudes, being listened to, and being able to trust those who provide care.^12^

It is important to make as explicit as possible the contribution made by registered mental health nurses to service user recovery.^13^ If no attempt is made to capture the contribution of mental health nursing to the quality care agenda^14^ and its importance in enhancing the service users’ experience, there is a risk of doing a disservice to the profession and those that it supports and cares for. The purpose of this project was to meet this goal and develop a tool to assess the effectiveness of the role of the registered mental health nurse in improving service user outcomes and the overall quality agenda of health‐care organizations.^15^ As far as the authors are aware, such a tool has not yet been developed; consequently, there is no way to measure the nature and effectiveness of “face‐time” therapeutic engagement. Therapeutic engagement itself and its impact on the quality of service user experience as perceived by them and the registered mental health nurses who deliver care have also not been measured.

An array of rating scales do exist to measure therapeutic engagement; however, either their tendency is to measure therapeutic engagement within research^16^ or they are qualitative in nature, therefore making it difficult to quantify the extent and quality of therapeutic engagement. In addition, these rating scales have been developed to assess the relationship between multidisciplinary clinicians and service users who experience mental illness. None of these scales, with the exception of the measure of service user perceptions of the design of inpatient wards,^17^ used psychometric methods,^18^ the foundation of measurement generation. The body of literature on mental health service user involvement in the development of measurement tools does indeed include a tool of service user perceptions and opinions of inpatient wards. Results from this study echo those of Chambers et al.^19^ who also identified that service users’ physical environment is important for recovery and that practitioners need time to engage with service user narratives surrounding “feelings of imprisonment” resulting from “a lack of privacy, being under constant supervision and all wants and/or needs for example ground leave having to be negotiated with staff.”^19^

Whilst some measures offer insights into therapeutic engagement and have been coproduced with service users,^17^ none of them assess one‐to‐one interaction between service users and registered mental health nurses on acute inpatient psychiatric settings as well as assessing the environment and atmosphere on the ward from both perspectives. It should be made clear that by assessing the environment and atmosphere, the authors denote the delivery of care within the therapeutic milieu of the clinical environment and not the physical environment itself. Examples include professional manner, verbal and non‐verbal communication and dignity and respect.

Whilst the authors recognize that colleagues have assessed the robustness of how psychiatric ward structures and design make an important contribution to patient outcomes and well‐being, our article focuses on the development of a psychometrically tested tool that captures the therapeutic engagement between nurses and service users (from both perspectives) which according to Peplau^8^ is at the core of quality mental health nursing. Like Csipke et al.,^17^ we demonstrate that service user perceptions are an important resource in the evaluation of inpatient psychiatric care and that the experiences of inpatient service users enable in‐depth information.

Our aim was to break new ground in that we would develop a short and simple “fit‐for‐purpose” tool^20^ which can be routinely used within acute inpatient settings to measure components of engagement from the perspective of the service users and the registered mental health nurses. The tool aims to identify and quantify the “invisible” nature of therapeutic interaction between registered mental health nurses and service users and provide robust monitoring of nursing activity and the engagement of service users in decision making about care and treatment. It can offer opportunity for transparency of activity and feed into the health‐care organizations’ other key performance indicators (KPI). Additionally, it will provide reassurance for directors of nursing about the nature and quality of nurses’ engagement and the degree to which they are aspiring to partnership working with service users as a means to enabling recovery.

Service user involvement will provide information through perception and opinion that may lead to beneficial changes in the services that they and others receive at present or in the future. Information from this study and the questionnaire itself will help to advise mental health nursing staff at all levels of seniority about the nature of therapeutic engagement experienced by service users. We are also hoping that the tool will tell us about service user involvement in the decision making and monitoring of their treatment and/or care to ensure it is offered with care, compassion, dignity and respect.^12^ Involvement of registered mental health nurses will offer opportunity to raise awareness within mental health nursing about therapeutic engagement in general.

## 2 DEVELOPMENT METHODOLOGY

All service users involved in this project were aged 18 years or over and had resided for more than 1 week within an adult acute inpatient mental health care setting (voluntary or detained), had mental capacity to consent to participate and had good command of the English language. Due to limited study resources, we were unable to provide translated versions of the documentation or to provide interpreters. It was made clear to the service users that their involvement would be meaningful (but that their role would be diverse) throughout the different stages of the project. Coproduction is the term that best describes service users’ level of involvement throughout the project. (In partnership, they helped to provide the care element for the tool as well as its design [and to help develop a mirrored nursing tool which would later be tested by their peers]). The number of service users involved and the level of their involvement depended on the stage of the project. At each stage of the design and development, service users were given structured activities and their views as to the clarity and accessibility of these were assessed as part of the study.

## 3 STAGES AND SERVICE USER ACTIVITIES

The TEQ was developed in collaboration with service users and registered mental health nurses; however, the focus of this article will be on the comprehensive involvement of service users.

In the following section, we describe the three stages of developing the tool as advocated by Nunnally and Bernstein.^18^ It should be noted that throughout this process, we consulted extensively with nursing staff; however, this article describes the involvement and views of service users in the development of the tool.

### 3.1 Statement generation stage

At this stage, the aim was to generate a statement pool for the tool in consultation with service users. A preliminary pool of statements for the tool were based upon themes extracted from the literature,^21^ and feedback from two sources: a therapeutic engagement workshop (n=70) involving service users and clinical nurse academics which was organized to address and specifically focus on the question “How can we measure nurses therapeutic engagement in a quantifiable way?” and from findings from the “Lived Experience of Detained Patients” project (in‐depth interviews with 19 detained service users).^19^ The latter methods provided service users with a platform whereby they could discuss their ideas and voice their concerns, thoughts and/or feelings. The data from these two sources were analysed thematically and the statements broken down into specific statements for inclusion in the tool. The tool was subsequently developed on the basis that therapeutic engagement is multifaceted. On combining these data sets, a service user version and a registered mental health nurse version were developed. Both versions include the same scoring scale and statements, but the wording is slightly different. Only the service user version is discussed in this article. Twenty‐five statements were generated through discussion within the research team for the tool. Each statement was measured on a 5‐point Likert scale,^22^ a psychometric scale commonly used in research that employs questionnaires with response choices ranging from strongly agree (1) to strongly disagree (5). Each statement was to be scored in relation to two aspects: the environment and atmosphere of the ward (to understand the overall therapeutic milieu), and 1:1 sessions with their service users named registered mental health nurse. All of the 25 statements referred to the present to capture current experience. No statements were presented in reverse format; all were positively phrased and written in language deemed by the research team to be familiar, plain and simple.

Reflecting the themes that emerged from the data analysis, the tool was split into five subsections (or themes) with each theme containing five statements. The five themes were identified by the research team as Compassion, Communication, Courage, Commitment and Collaboration, four of which overlapped with the “6Cs” of nursing.^23^ Each of the themes included sets of statements which reflect specific areas of therapeutic engagement and considered important to service users.

Upon review by an “expert” clinical and academic mental health nursing panel which included service users, the following revisions were made to the service user version of the tool. The service users on the expert panel described the need for service user participants to take “ownership” of the statements in the tool and to be “bothered” about the impact of their answers. As a result, the panel agreed that the statements should be personalized, so the word “me” or “I” was incorporated into each of the service user statements. The number of statements was reduced from 25 to 20; this was deemed necessary to reduce the “burden” to the service users completing the tool, for example completion time and repetition. The Likert scale used for response choices within each given theme was reduced from 5 to 4. A 4‐point Likert response scale was chosen to “force” respondents to agree or disagree with each statement.^24^ The five subsections (and their visible titles) remained, but now each encompassed four instead of five statements.

### 3.2 Pre‐testing stage

At this stage, the aim was for the service users to review the preliminary statement pool. Pre‐testing is crucial for identifying problems with a tool, that is the TEQ at an early stage.^18^ This included the wording and content of the statements, which can cause confusion with the overall meaning of a statement as well as misinterpretation of individual terms or concepts,^25^ clinical appropriateness, the clarity of instructions, statement‐stems and the Likert response options. The TEQ appears to be sensitive to the responses of participants, hence the importance of having service users reviewed it at this stage of the development process.

Service user members of an Education and Research Group (n=12) connected to the university department in which two of the authors work were invited to test and give feedback on the TEQ. The group members were of mixed gender, age and ethnicity and either working within a mental health setting, for example research, or residing in an inpatient setting. The Education and Research Group service user participants were asked to individually complete the preliminary 20‐statement questionnaire in the presence of a service user researcher and a group facilitator for support if needed. The service user researcher was recruited for just this task and was not part of the research team. Following completion, the service users were asked to participate in a focus group to share feedback regarding clarity of instructions, statements, scoring method and response options, use of language/wording, ease of completion, presentation/layout and anything they felt could be improved and/or changed. In addition to completing the TEQ, respondents were asked to rate the overall impression of the TEQ on a 5‐point Likert scale: 1 (Very dissatisfied) to 5 (Very satisfied). Three qualitative statements followed with regard to the TEQ's strengths, any improvements needed to be made and lastly the sharing of any general thoughts about the questionnaire. Responses to these qualitative statements were completed on a feedback sheet. The completed project documents were placed in a sealed envelope by each respondent for collection by the service user researcher, who then forwarded them to the project's principal investigator.

To capture the feedback, a focus group was chosen as the forum in which to “share” feedback and provide opportunity for peer support, exchange of ideas and common values.^26^, ^27^ The focus group adopted an informal, unstructured discussion, using the “think‐aloud” approach,^28^ in order for the service user respondents to feel comfortable in describing their thoughts about the questionnaire. This method avoided interpretation by the service user respondents and only assumed a very simple verbalization process which allows for discussion to be considered as objective data.^28^ Guidance questions were formulated and used as prompts in the focus group which lasted approximately 25 minutes. The focus group was not audio‐ or video‐recorded; however, the service user researcher took notes as appropriate.

Feedback from the service users included the following: it was considered a good idea to have a tool like the TEQ; at first glance, pages seemed crowded due to the titles of the themes/sections, and therefore, the pages gave the impression of being time‐consuming to complete; helpful to have the 1‐4 scoring key on every page for quick reference; the print in a bigger font and remove section titles. The service users liked the use of “me” or “I” in the statements—“The use of ‘I’ in the statements made me feel individualised and invited me to take ownership of what I was reading.” All the phrases were noted to be positively phrased, for example “Nurses respect my time and personal space and make me feel at ease” and “The relationship that I have with my identified ‘nurse’ is a caring one.” The consensus was that service users liked this positive approach. Some considered that the statement about the future was emotive and might upset some service users; however, it was retained as it links to advance directives and recovery principles.^29^ For some, this statement was ambiguous, calling into question whether it refers to health care or life. The focus group decided that it meant health care. The context for completing the questionnaires was said to need better explanation, and some service users felt self‐conscious about completing it, verbalizing their hesitance as being related to judging specific nurses who may see the questionnaire's content. The “rules” of anonymity and confidentiality were reiterated by the facilitator and service user researcher which seemed to put the service users at ease. To avoid such issues/concerns whilst completing the questionnaire, the group facilitator and service user researcher suggested having a peer‐fieldworker on the ward who could guide them. This would help mitigate anxiety about anonymity, confusion over instructions and unwelcome emotions and would provide encouragement. The facilitator also felt that service user data are more authentic if forms are completed with the aid of peer‐fieldworkers rather than clinical staff. Some of the service users considered that if unwell, they may not “feel up‐to” completing the form and would need “encouragement,” suggesting it would depend on how it was “sold” to them.

In summary, the statements met with general approval from the service user participants; suggested changes were made with respect to instructions, layout and wording, with some appropriate alternatives offered.

The feedback data from the service users were collated and noted. No new concepts/themes of importance and/or relevance manifested and no additional statements suggested. The service users were found to be reflective in their responses to the qualitative questions, and the feedback sheet appeared to be a platform for verbalizing their thoughts and feelings about being on an inpatient unit/ward. In general, service users were very complimentary about the questionnaire: “Simple to answer questions. Didn't feel overwhelmed trying to answer the questions,” “It was simple, straight to the point, not long winded,” “It makes you think about the time on the ward and different aspects of your care,” “It was understandable, I wouldn't make any alterations,” “The questionnaire was well constructed from start to finish,” “Has the ability to cover all aspects of my care,” and “The questionnaire matches what I am experiencing. The tool will give a good idea of the quality of nursing care.” Service users expressed high satisfaction with the TEQ by rating their satisfaction as 4 or 5 out of 5, on a 5‐point Likert scale (Overall, how would you rate the TEQ?), where 5 was the most satisfied. The TEQ includes domains/themes and a response scale that has been determined through consultation with service users and is therefore meaningful to them.

### 3.3 Statement reduction stage

Following feedback and revisions, the tool retained the 20 statements. The 20‐statement revised tool became the basis for a much larger project involving service users from four Mental Health NHS Trusts across England. Service users (n=86) completed the revised questionnaire within their care (acute inpatient) environment. The data were analysed by a statistician, and it appears that two groups or factors are formed in the TEQ—care delivery and care interactions. Example questions include “(The nursing staff…) Show me respect at all times, Give me support at all times, Accept me for who I am,” in the “environment and atmosphere of the ward as created by the nursing staff” part of the questionnaire, and “(My named nurse…) Works in partnership with me to achieve my goals, Promotes caring relationships, Supports me in the choices that I make,” in the 1:1 sessions with my named nurse section. The TEQ's psychometric properties were reviewed, and it was found that the TEQ behaves well as an assessment scale. Information about the groups/factors of the TEQ and its psychometric properties will be discussed in another article pertaining to this project in the near future.

## 4 DISCUSSION

The aim of this project was to develop a measure of therapeutic engagement that combines the service users’ perspective with that of the registered mental health nurse. Adopting a model for the development of a measure is important as the results of studies surrounding therapeutic engagement are dependent on the quality of the tools used for data collection.^3^ The research team have structured the TEQ to be multidimensional with themes of Compassion, Communication, Courage, Commitment and Collaboration as the facets underlying this 20‐statement tool (to be described in a subsequent paper).

There is a growing literature on the variables that are associated with therapeutic engagement.^30^, ^31^ It is important to identify the many variables that can contribute to sound therapeutic engagement in mental health settings. Patient outcomes are varied and dependent upon an assortment and mixture of factors.^32^ Identifying these factors is helpful to service users and health and social care professionals alike in guiding interventions and forming a basis for clinician‐patient relationships.^33^ Identifying such variables will be significant in practice as it may help professionals to target education and training of staff in relation to those predictions.^34^, ^35^

Service user involvement in research and educational programmes for staff is advocated, but the realities of inclusion and engagement of this group are not always easy.^36^, ^37^ Nevertheless, their contribution is valued. If research is the route to good practice, then researchers should involve the vulnerable, the stigmatized and the marginalized and be vigilant about the research process.^38^

Participation of service users in this project was a major factor in its success. Their input contributed to the content and design of the TEQ which in turn saw the development of a service‐user‐friendly and clinically appropriate measure. All too infrequently service users are involved in tool development and design, but the learning to be had from their involvement in this project is important. Patient experience narratives led to the development of a tool that has potential benefits for the care and treatment delivered. Such information may help to better plan and allocate health and social care resources.^39^ The information and feedback service users provide presents a realistic, patient‐centred and “lived” representation;^39^ the results have sound face and content validity. Metrics underpin clinical research and clinical practice; therefore, if service users can be involved in the content and design of these assessment tools, they are more likely to be clinically appropriate and ethically sound to those who access services.

## 5 STRENGTHS AND LIMITATIONS OF THE PROJECT

This project shows that including a small group of service users in research can generate change. Service user perspective brought benefit to the TEQ, for example the way questions were phrased and presented. Likewise, the personal experiences of service users provided insight into what they felt was important with regard to their relationships with nursing staff. Involving service users in the design of the tool ensured that it is relevant to the needs of those who will be completing the tool in the future. Service users provided useful feedback which enabled their “voice” to be heard and their opinion on what they believe is good practice and/or what concerns them to be counted.^40^ Working in partnership with service users can help to develop ways of working that improve service quality. The active involvement of service users in research enables them to develop a sense of empowerment and provides opportunity to share and allow others to benefit from their unique experience by engaging service users.^7^, ^41^ If health and social care research is to be of real benefit to service users, then we must strive to involve them more in setting the questions to which we are seeking answers; time and time again evidence has demonstrated that service user involvement results in outcomes that are more relevant and useful to the practice that is delivered.^4^ Strength of this project derives from the collaboration with service users at each stage of the development of the tool.

This project sought to present important issues surrounding interpersonal activity between registered mental health nurses and service users; even so, the authors are aware that other important concerns/issues of service users and staff may have been omitted, and therefore, the tool may not be all‐encompassing. The project relied upon participant self‐report, and a substantial literature exists concerning the numerous problems of self‐report data, for example issue of social desirability.^42^, ^43^ Inclusion of other sources, for example service user notes, and having service users as part of the research team would have been beneficial in the development of the TEQ.

We anticipate that data gathered by the TEQ will provide information about the nature of therapeutic engagement between service users and registered mental health nurses in acute inpatient environments. In addition, we hope that the TEQ will inform about service users’ involvement in the decision making and monitoring of treatment and/or care and how it is offered.

## ETHICAL CONSIDERATIONS

Ethical approval was obtained from the NHS National Research Ethics Service (NRES) prior to the project commencing. The project was registered with the Research and Development Committee for the Mental Health Trusts involved in the research. The nature and objectives of the project were explained to all potential participants. Written informed consent was obtained from each participant prior to data collection. All participants were assured of their confidentiality and their right to withdraw from the project without penalty.

## CONFLICT OF INTERESTS

The authors declare no known conflict of interest.
